# Does TLS Exist in Canine Mammary Gland Tumours? Preliminary Results in Simple Carcinomas

**DOI:** 10.3390/vetsci9110628

**Published:** 2022-11-11

**Authors:** Giada Giambrone, Stefania Di Giorgio, Cecilia Vullo, Gabriele Marino, Roberto Puleio, Francesca Mariotti, Giuseppe Mazzullo, Alessandra Sfacteria

**Affiliations:** 1 Department of Veterinary Sciences, University of Messina, Via G. Palatucci, 98168 Messina, Italy; 2 Department of Chemical, Biological, Pharmaceutical and Environmental Sciences, University of Messina, Viale Ferdinando Stagno d’Alcontres 31, 98166 Messina, Italy; 3 Histopathology and Immunohistochemistry Laboratory, Istituto Zooprofilattico Sperimentale della Sicilia, Via Gino Marinuzzi 3, 90129 Palermo, Italy; 4 School of Biosciences and Veterinary Medicine, University of Camerino, Via Circonvallazione, 93, 62032 Camerino, Italy

**Keywords:** mammary gland, tumour, TLSs, tertiary lymphoid structures

## Abstract

**Simple Summary:**

Studies on the tumour microenvironment show that part of the body’s defence against neoplastic cells is carried out directly in the tumour through the organization of immune cellular aggregates, called tertiary lymphoid structures (TLSs). This study demonstrates the existence of TLS in canine mammary simple carcinomas, where some inflammatory infiltrates assume a follicle-like organization, especially in high-grade carcinomas and simulate the follicle of secondary lymphoid organs (SLOs). The role of TLS is controversial and it has been suggested that they contribute to an immunosuppressive environment or, on the contrary, have positive effects on neoplasm growth. Although canine mammary tumours have long been proposed as a model for studying breast cancer, there is still little research on the tumour stromal microenvironment in veterinary medicine and TLS evidence could allow for advances in understanding the process of tumour immunoediting.

**Abstract:**

Neoplastic progression is influenced by the expression of tumour antigens that activate an anti-tumour immune response. Human medical studies show that this body defence is carried out in secondary lymphoid organs (SLOs) but also directly in the tumour through organized cellular aggregates that are called tertiary lymphoid structures (TLSs). However, their occurrence has different meanings in different tumour types. For example, the presence of TLSs in breast cancer is associated with the most aggressive subtypes. This paper aimed to study TLSs in canine mammary simple carcinomas. A morphological assessment of the inflammatory infiltrate was performed on H&E sections of fifty cases. Immunohistochemistry was then carried out to typify the inflammatory cells in the tumour microenvironment. Results showed that, sometimes, inflammatory infiltrates were organized in follicles close to high-grade carcinomas, simulating a lymphoid organization, as in breast cancer. Therefore, we can assume that even in canine mammary tumours, TLSs exist and they are entities to consider due to their presence in the most aggressive histotypes or tumours with a high degree of malignancy.

## 1. Introduction

Neoplastic immune surveillance is due to a series of genetic alterations, resulting in the expression of tumour antigens that activate an adaptive anti-tumour immune response committed to eliminating cancer cells. Typically, the adaptive immune response takes place in secondary lymphoid organs (SLOs), in which dendritic cells present molecule–peptide complexes to CD4+ T and CD8+ T cells. In SLOs, B lymphocytes, following antigen presentation, are activated in primary follicles, then secondary follicles, which eventually become germinal centres. This results in the proliferation and differentiation of T lymphocytes and memory B cells that migrate into the tumour and lead to the destruction of the neoplastic cells [1]. However, human medical studies on the tumour microenvironment show that the body’s defence against neoplastic tissue is not only carried out in secondary lymphoid structures, but also directly in the tumour through the organization of cellular aggregates that mimic the behaviour of SLOs and are called tertiary lymphoid structures (TLSs) [2]. They are the result of a continuous and prolonged exposure of the tissue to inflammatory signals and have been described in various pathophysiological conditions, such as autoimmune diseases, infectious diseases, organ transplants, inflammatory disorders and neoplastic processes [3,4]. In the human literature, TLSs have been found in the stroma, margins and central portion of different tumour types. They are generally composed of T-lymphocyte-rich areas, with dendritic cells juxtaposed to B-lymphocyte follicles with characteristic germinal centres and surrounded by plasma cells. Some high endothelial venules, such as those found in SLOs, can be seen nearby and are intended to allow for the passage of lymphocytes [5]. However, there are studies suggesting that different stages of maturation of TLSs should be considered [6,7]. Therefore, the study of the TLS structure shows that TLSs represent a site for the presentation of tumour antigens close to dendritic cells, resulting in T and B cell activation, proliferation and differentiation and, thus, both a cell-mediated and humoral immune response [8].

The density of TLSs correlates with the density of CD4+ T and CD8+ T cells in the tumour and the detection of tertiary structures has been associated with a favourable prognosis for many solid malignancies, probably related to their ability to induce a durable systemic anti-tumour response [9].

Dendritic cells (DCs) present antigen to CD4+ T-cells, but studies in ovarian metastases of breast cancer have shown that DCs also appear to be involved in antigen presentation to germinal centre B-cells. Furthermore, locally produced anti-tumour immunoglobulin G increases the ability of dendritic cells to present antigen via IgG-specific receptors [10].

In general, studies have shown that TLSs can generate a response from effector and memory B cells by producing antibodies against tumour antigen and protecting against metastasis [11]. The link between TLS and histopathological parameters is quite controversial and seems to have different implications depending on the type of neoplasm considered. In breast cancer, high-grade tumours were found to be twice as likely to show TLSs as low-grade tumours [12].

In general, the presence of TLSs is associated with the most aggressive breast cancer subtypes, such as TNBC (triple-negative breast cancer), HER2 enriched and luminal B, while tumours without TLSs tend to be luminal A [13].

In HER2- breast cancer, it has been seen that the presence of tumour cells inside the TLS is associated with lymphatic invasion and lymph node involvement [14]. It could be hypothesised that tumour cells penetrating TLSs more easily metastasise to regional lymph nodes [15].

Although canine mammary tumours have long been proposed as a model for studying breast cancer, there is still little research on the tumour stromal microenvironment in veterinary medicine compared with human medicine. There are already several studies investigating the role of various individual inflammatory populations, but no study has highlighted the presence of lymphoid tertiary structures in dog mammary tumours. The aim of this study was to investigate the immune cell infiltrates in simple carcinomas of the canine mammary gland and give evidence of TLS structures in order to allow for advances in understanding the processes of tumour immunoediting.

## 2. Materials and Methods

Fifty cases of mammary gland lesions were retrieved from the archives of the Unit of Veterinary Pathology of the Department of Veterinary Sciences of Messina. All the cases were re-classified according to the most recent classification [16]. We included in the study forty simple mammary carcinomas and ten cases of hyperplastic or dysplastic lesions. The neoplastic histotypes were then assigned to a grade of malignancy according to the histological criteria provided by Peña et al. (2013) [17] (Table 1).

On hematoxylin and eosin (H&E) sections, a careful morphological assessment of the inflammatory infiltrate was carried out. Immunohistochemistry was then performed to typify inflammatory cells and molecules of interest in the tumour microenvironment. Samples were fixed in formalin and embedded in paraffin. Then, 5 µm sections on poly-L-lysine-coated slides underwent antigen unmasking by the means of a microwave steaming in citrate buffer at pH 6 or EDTA at pH 8. Hydrogen peroxide was used to block the endogenous peroxidases and a blocking reagent to block non-specific protein reactions (ChemCruz—UltraCruz Blocking Reagent, Santa Cruz Biotechnology). The sections were then incubated with the selected primary antibodies (as reported in Table 2), overnight at 4 °C. As revelation method, biotin- or HRP-conjugated secondary antibodies were then applied followed by an incubation with acetylated Streptavidin (Biospa antibiotic products, Milan; for biotin-conjugated antibody only). Vector Vip or DAB enzyme substrate, followed by nuclear counterstain with Haematoxylin or Light Green, were used to stain the immune reaction.

Each round of IF or IHC included negative reagent controls and internal controls. Each antibody was tested under different conditions as probing with and without AR, detection systems with biotin and HRP-conjugated secondary Abs. The use of multiple antibodies of the same isotype and similar concentrations represented a set of irrelevant reagent controls. Moreover, to exclude DAB false positivity to immune cells, Vector Vip was used as alternate chromogen. IF for IgG was performed testing two direct Abs different for the brand, clone and species of origin.

Immunohistochemistry positivity was assessed as membranous or cytoplasmic.

## 3. Results

**Figure 1:**
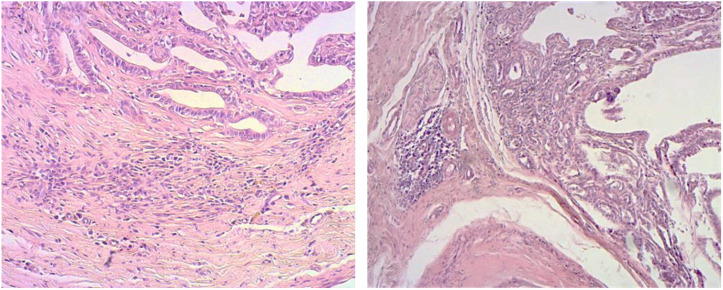
Inflammatory infiltrate in the stromal areas of tubulopapillary carcinoma (grade I) with the appearance of bands and aggregates.

**Figure 2:**
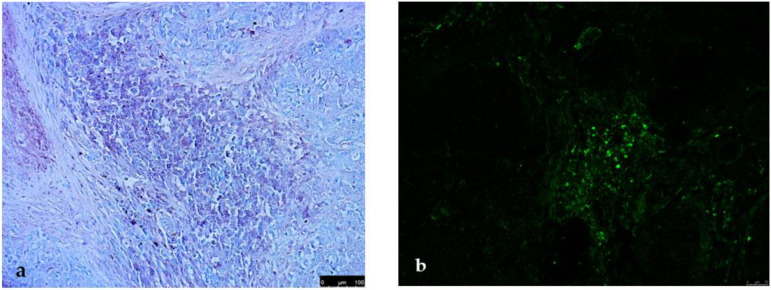

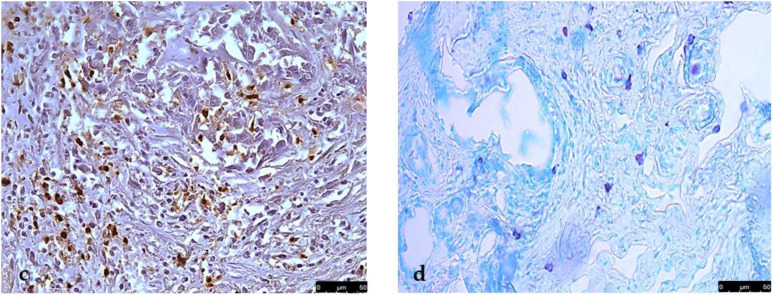
Stromal infiltrates mainly composed of T lymphocytes ((**a**), CD3, Vector Vip, 5×) with a minimal representation of plasma cells ((**b**), IgG, FITC, 10×). Macrophages, numerous, scattered and independent of the lymphoplasmacytic infiltrates ((**c**), MAC/387, DAB, 20×). Mast cells, in hyperplastic/dysplastic areas ((**d**), MC tryptase, Vector Vip, 10×).

Comparative evaluation of the different histotypes showed that inflammatory cells were invariably present in the peritumoural stroma. The cell types were mainly mast cells and macrophages in areas of hyperplasia/dysplasia, whereas lymphocytes and plasma cells predominated in the neoplastic areas. In simple carcinomas, the infiltrate in the stromal areas was uniformly distributed close to the vessels and the neoplastic epithelium, in some cases, with the appearance of bands and in others as more conspicuous aggregates (Figure 1). Immunohistochemistry with CD3, specific for all T lymphocytes, showed that the stromal infiltrates were predominantly T lymphocytes with a minimal representation of IgG-positive cells (Figure 2a,b). Macrophages, marked with macrophage markers, were numerous but scattered throughout the peritumoural and intratumoural sites and independent of the lymphoplasmacytic infiltrates (Figure 2c).

Mast cells, detected by mast cell tryptase, were present in greater numbers in hyperplastic/dysplastic areas and along the margins of invasion of malignant neoplasms (Figure 2d).

**Figure 3:**
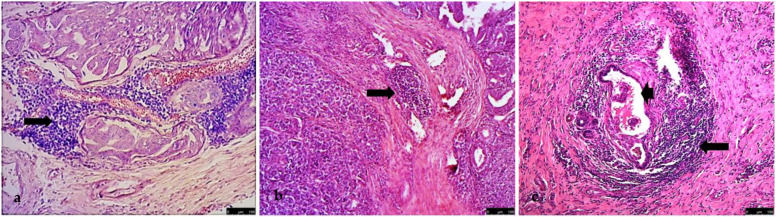
In high-grade carcinomas, (**a**) tubulopapillary carcinoma (10×) and (**b**) solid carcinoma (10×), inflammatory infiltrates with a follicle-like disposition: numerous lymphoid-like cells assumed a concentric pattern around intact vessels (arrow) or (**c**) vessels containing neoplastic emboli (arrowhead, HE, 10×).

Only in high-grade carcinomas, in the peritumoral stroma, inflammatory infiltrates were sometimes found with a different, follicle-like disposition: numerous lymphoid-like cells assumed a concentric pattern around intact vessels or vessels containing neoplastic emboli (Figure 3).

**Figure 4:**
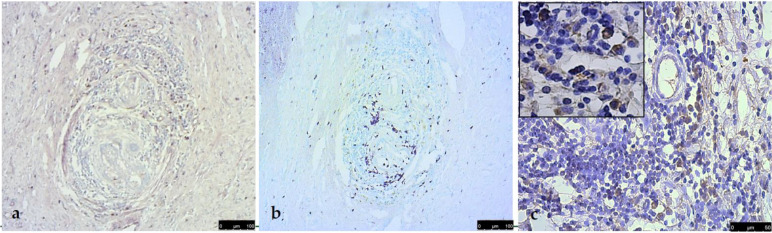
Immunohistochemistry of follicle-like structures: mast cells ((**a**), MC tryptase, Vector Vip, 10×); macrophages ((**b**), MAC/387, Vector Vip, 10×). Follicular dendritic cells (FDCs) variously located in both the outer and inner parts of the structure ((**c**), CD21, DAB, 20×; inset, 40×).

The immunohistochemical investigation of these cases showed that mast cells were less represented, within the follicular structures, but constantly mixed with macrophages and in a perivascular location (Figure 4a). Macrophages were represented and in the areas closest to the vessels (Figure 4b). The use of CD21 revealed the presence of several follicular dendritic cells (FDCs), variously located in both the outer and inner parts of the structure (Figure 4c).

**Figure 5:**
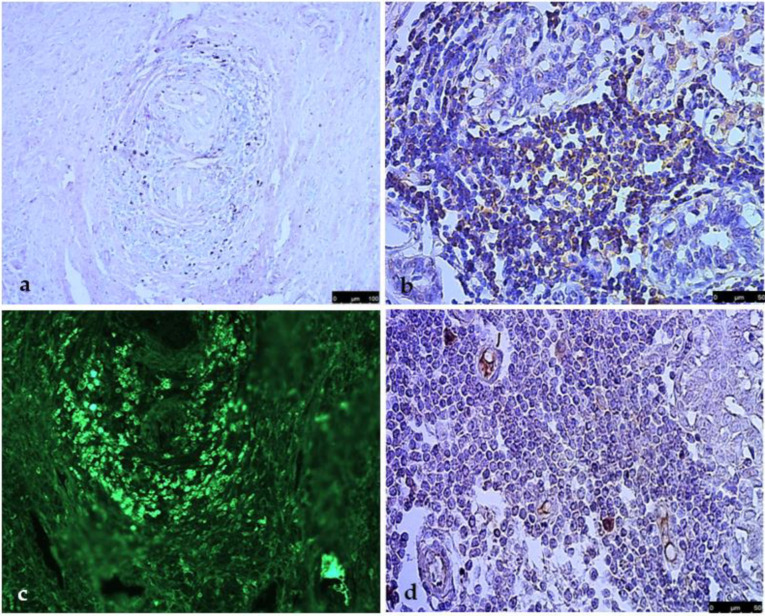
In the follicle-like structures, T lymphocytes are mainly located in the outermost areas in a concentric pattern ((**a**), CD3, Vector Vip, 10×), CD20+ cells are in the inner part ((**b**), DAB, 20×). The remaining inflammatory population reveal an intense positivity for the IgG marker ((**c**), FITC, 20×). The presence of vascular structures such as HEVs is confirmed by positivity for MECA-79 ((**d**), DAB, 20×).

T lymphocytes sought by CD3 were located predominantly, but not exclusively, in the outermost areas in a concentric pattern (Figure 5a). Instead, immunohistochemistry with CD20 showed high positivity concentrated in the inner areas (Figure 5b). The remaining inflammatory population revealed an intense positivity for the IgG marker and was assumed to be plasma cells (Figure 5c). In addition, the presence of vascular structures, already identifiable histologically as high-endothelium venules (HEVs), was further confirmed by their positivity for MECA-79 (Figure 5d).

## 4. Discussion

The results showed that the immune cells had a typical perivascular localization. The composition of the infiltrate was variable according to the mammary lesion histotype, with a proliferation of conspicuous infiltrates close to malignant epithelial proliferations where the lymphocytic and macrophagic cells took over according to Sfacteria et al. [18]. As already reported by Estrela-Lima et al. [19] and Carvalho et al. [20], the density of T lymphocytes was higher than that of plasma cells in most inflammatory infiltrates. In contrast, the results showed that in areas morphologically similar to follicular lymphoid structures, the situation was the reverse, as IgG-positive cells were in the majority. Macrophages were well represented and macrophage marker positive.

The latter is known to mark M1-polarised macrophages and, knowing their anti-tumour role, it could be assumed that they are still part of the body’s attempt to combat neoplastic invasion or that they represent phenotypes that will subsequently be converted to M2 by the tumour microenvironment. The first hypothesis could be better confirmed by the macrophage distribution, which was scattered and not linked to the lymphoplasmacytic inflammatory infiltrate. However, the most characteristic forms of tertiary lymphoid structures were only found in the most malignant histotypes, with localization around emboli or vessels. The concentric pattern simulated a lymphoid organisation similar to that described in breast cancer [5]. The composition of these formations was mainly plasma cells, B cells, T lymphocytes, macrophages and mast cells in order of highest representation. Although the organisation of these structures did not fully reflect a cellular distribution as that of secondary lymphoid structures, it is now recognised that TLSs are dynamic entities, with varying organisation from simple clusters of lymphocytes to more complex structures, such as SLOs [21,22]. In fact, even today, in human medicine, there is no full agreement on the cell components that define a TLS. However, it is recognized that there are three different stages of maturation. The first stage is the least organized and consists of T and B lymphocyte aggregates without the presence of FDCs. Primary follicle-like TLSs contain FDCs, but no germinal centres are present. Finally, fully mature secondary follicle-like TLSs also have germinal centres [23]. A common constant in these different arrangements is the distribution in the vicinity of high-endothelium venules, precisely because of their role in involving the various cellular components that become part of TLSs [9,10,11,12,13,14,15,16,17,18,19,20,21,22,23,24]. The inflammatory infiltrates we identified were always placed near structures identifiable as high-endothelium venules, both histologically and immunohistochemically with MECA-79. Furthermore, in areas with concentric-like patterning, CD3 + T lymphocytes were mainly placed in the outer layer, surrounding an area rich in CD20 + B cells and plasma cells. Again, it was also possible to detect CD21+ FDCs, variously displaced in the structure. Thus, from what has been shown, we can say that tertiary lymphoid structures at different stages of maturation can also be present in canine mammary tumours. T-lymphocyte-rich aggregates can be considered an early stage of maturation and called lymphoid aggregates or TILs (tumour lymphocyte infiltrates). In follicle-like structures, the orderly arrangement of individual cell populations and the presence of FDCs allows one to recognise the second stage of maturation (primary follicle). 

The high positivity for IgG, correlated with the malignancy of the histotype analysed, further confirms what was already highlighted [18] and finds agreement with what has been reported in human medicine. Indeed, there seems to be a correlation between IgG expression and histological subtypes of carcinoma, since poorly differentiated breast cancer cells express more IgG than well-differentiated ones [25]. The above considerations support the hypothesis that humoral immunity is involved in tumour progression. In addition, another consideration is that the mast cells, although poorly represented, could be directly recruited and conditioned by the immunoglobulins. Indeed, large amounts of IgG can bind mast cells and prevent their degranulation, since these cells, in addition to binding IgE, also express receptors for the Fc portion of IgG. Mast cells can, therefore, undergo antibody modulation [26]. Groot et al. [27] demonstrated that FLCs (immunoglobulin-free light chains) activate mast cells, resulting in the release of molecules that induce tumour growth and suggested that increased expression of FLCs correlated with poor survival.

Regarding macrophages, the M1 type was always detected, so their role is questionable for the reasons already given above. However, another possible interpretation may be related to the role that M1 macrophages play in leading to the formation of TLSs. In fact, M1 cells and B lymphocytes can replace lymphoid tissue inducer cells (LTi cells), responsible for triggering the events that lead to the formation of TLSs [28,29].

In the present study, the overall prognostic role of TLSs can be only speculated because of the unavailability of the follow-up for the analysed samples. However, based on the higher representation of TLSs with a more defined organisation, in high-grade tumours close to the areas of infiltration and around neoplastic emboli, it can be suggested that dog tertiary lymphoid structures could also reflect a worse prognosis, as already highlighted in breast cancer by Sofopoulos et al. [30]. It is clear from the above that if TLSs are the main site where anti-tumour immune response takes place, it is important to assess their impact on treatment response and their modulation by therapies [11]. Indeed, the possibility of developing strategies targeting the neogenesis of TLSs in tumours to enable the development of memory B and T lymphocytes and ensure optimal defence against neoplastic tissue has been considered. The HEVs surrounding TLSs allow lymphocytes to enter the tumour, so a therapeutic strategy could be aimed at improving this feature [20].

Looking more closely at the prognostic impact of tertiary structures in breast cancer, the presence of peritumoural TLSs is associated with grade 3 tumours [30]. The poor prognosis depends on both the location of the TLSs and the density. In HER2+ tumours, it was seen that the presence of TLSs in the invasive and/or peritumoural margin was related to better disease-free survival but worse overall survival, whereas this difference is not detected for HER2- tumours [14].

Some studies suggest that inflammation-associated TLSs serve as niches for tumour progenitor cells and may lead, for example, to a recurrence of hepatocellular carcinoma. Peripheral TLS may, thus, contribute to an immunosuppressive environment, supporting tumour growth through negative effects on anti-tumour immunity or positive effects on the neoplasm itself [15].

## 5. Conclusions

The tumour microenvironment is a dynamic entity characterised by the involvement of chemokines and immune cell populations that can be modulated by the tumour for its own survival and progression. In general, from what has been observed, we can, however, state that also in canine mammary tumours, we can speak of tertiary lymphoid structures with various degrees of organisation. The prognostic role of TLSs in dogs can, however, only be speculated on, as actual post-treatment follow-up would be required. Despite this, their greater representation in more malignant histotypes suggests that TLSs might also have a negative prognostic value in dogs. However, even though these are preliminary studies to be accompanied by further verification and research, they are fully in line with the concept of translational medicine, especially considering that the mammary tumour in dogs has long been considered for a model for female breast cancer. All this also opens the possibility of identifying new diagnostic and predictive markers and even new therapeutic targets.

## Figures and Tables

**Table 1:** List of cases considered (n.a. not available).

Case	Breed	Age	Histotype	Grade
1	Sicilian hound	7	Tubular carcinoma	I
2	Crossbreed	n.a.	Tubular carcinoma	I
3	Crossbreed	12	Tubular carcinoma	I
4	Jack russel	9	Tubular carcinoma	I
5	Crossbreed	n.a.	Tubular carcinoma	I
6	Crossbreed	n.a.	Tubular carcinoma	I
7	Chihuahua	8	Tubular carcinoma	I
8	Beagle	10	Tubular carcinoma	II
9	Crossbreed	11	Tubular carcinoma	II
10	Yorkshire terrier	7	Tubulopapillary carcinoma	I
11	Crossbreed	5	Tubulopapillary carcinoma	I
12	German shepherd	11	Tubulopapillary carcinoma	I
13	Yorkshire terrier	10	Tubulopapillary carcinoma	I
14	Beagle	8	Tubulopapillary carcinoma	I
15	Yorkshire terrier	4	Tubulopapillary carcinoma	II
16	Cocker spaniel	15	Tubulopapillary carcinoma	II
17	Cocker spaniel	10	Tubulopapillary carcinoma	II
18	Rottweiler	9	Tubulopapillary carcinoma	II
19	Crossbreed	9	Tubulopapillary carcinoma	II
20	Crossbreed	n.a.	Tubulopapillary carcinoma	II
21	Breton	8	Micropapillary carcinoma	II
22	Shih-Tzu	10	Micropapillary carcinoma	II
23	German shepherd	5	Micropapillary carcinoma	II
24	Jack russel	5	Micropapillary carcinoma	II
25	Jack russel	7	Micropapillary carcinoma	II
26	German shepherd	10	Micropapillary carcinoma	II
27	Yorkshire terrier	7	Micropapillary carcinoma	III
28	Dalmation dog	8	Micropapillary carcinoma	III
29	Crossbreed	n.a.	Micropapillary carcinoma	III
30	Pitbull	9	Micropapillary carcinoma	III
31	Golden retriever	8	Solid carcinoma	II
32	Crossbreed	10	Solid carcinoma	II
33	Crossbreed	14	Solid carcinoma	II
34	Crossbreed	12	Solid carcinoma	II
35	Shar pei	6	Solid carcinoma	II
36	Chihuahua	9	Solid carcinoma	II
37	Jack russel	10	Solid carcinoma	II
38	Crossbreed	n.a.	Solid carcinoma	III
39	Crossbreed	8	Solid carcinoma	III
40	Rottweiler	9	Anaplastic carcinoma	III
41	Pomeranian	13	Hyperplasia/Dysplasia	
42	Crossbreed	9	Hyperplasia/Dysplasia	
43	German shepherd	n.a.	Hyperplasia/Dysplasia	
44	Crossbreed	6	Hyperplasia/Dysplasia	
45	Yorkshire terrier	5	Hyperplasia/Dysplasia	
46	Poodle	10	Hyperplasia/Dysplasia	
47	Crossbreed	n.a.	Lobular Hyperplasia with Atypia	
48	Siberian husky	10	Hyperplasia/Dysplasia	
49	Poodle	6	Lobular Hyperplasia with Atypia	
50	Siberian husky	8	Hyperplasia/Dysplasia	

**Table 2:** Primary and secondary antibodies used for HIC and IF.

**Antibody I**	**Clone**	**Specificity**	**Dilution**	**Brand**
Mast cell Tryptase	10D11	Mast cells	1:100	Santa Cruz Biotechnology
Macrophage Marker	MAC/387	Macrophages	1:200	Santa Cruz Biotechnology
CD3		T lymphocytes	1:50	Santa Cruz Biotechnology
CD20	D-10	B lymphocytes	1:200	Santa Cruz Biotechnology
CD21	A-3	Follicular dendritic cells (FDCs)	1:200	Santa Cruz Biotechnology
MECA-79	MECA-79	High endothelial venules (HEVs)	1:200	Santa Cruz Biotechnology
Sheep Anti-canine IgG:FITC	OBT4041F	Immunoglobulin G dog	1:50	Oxford Biotechnology
Mouse Anti-human IgG:FITC	HP-6017	ImmunoglobulinG human	1:50	Sigma
**Antibody II**	**Clone**	**Specificity**	**Dilution**	**Brand**
Goat anti-mouse IgG-B		Mouse IgG	1:100	BioSpa, Milan
Donkey anti-goat IgG-B		Goat IgG	1:200	Santa Cruz Biotechnology

## Data Availability

Not applicable.
